# Indents

**Published:** 1918-09

**Authors:** 


### INDENTS

**☞Brief Articles of Technical or Scientific Value will receive notice and comment. Contributions invited.**

---

*Advantages of the Gold Inlay*

*First.* An opportunity to fulfill, in a larger degree, our ideas of extending cavity outlines to safe limits for permanency of work and, at the same time, conserving the strength of the tooth.

*Second.* The possibility, previously difficult, but now most simple, of outlining occlusal portions of restorations in metals to conform in full measure to the demands of utility as exemplified in nature.

*Third.* A protection to the pulp from irritation by the inter-position of cement.

*Fourth.* A relief from strain to patient and operator by lessened concentration in cases of large restorations.

*Fifth.* The reduction in the numbers of crowns by the broad application of the inlay.

Notwithstanding these advantages, a marked disadvantage has appeared through the tendency, and a very prevalent one, of using the inlay where one of the other methods would have given superior results and often at a considerable saving of time.

It also seems to have produced to some extent shiftlessness in procedures.—Dr. Walls, in *Journal N. D. A.*

*Ventilation in Schoolrooms*

Working in cooperation with the New York State Commission on Ventilation, whose investigations have been recorded from time to time in *The Journal,* the Bureau of Child Hygiene of the New York City Department of Health has contributed some striking evidence on the subject. In a comparison involving 5,533 pupils in many classrooms, the relation of the temperature and type of ventilation of the schoolrooms to absence due to respiratory disease was investigated. It was found that children in classrooms with closed windows and ventilated by mechanical methods, such as the plenum fan system, were more subject to such ailments severe enough to keep them from school attendance than were children who were in classrooms kept at the same or lower temperature and ventilated wholly by open windows. Furthermore, in the closed window, mechanically venilated type of room, the rate of respiratory diseases occurring among pupils in actual attendance, that is, not ill enough to be kept at home, was ninety-eight per cent. higher than in naturally ventilated schoolrooms at the same temperature (about sixty-eight F.), and about seventy per cent. higher than in the open window type of classroom kept at about 50. There was no difference between the sexes. Contrary to popular notions, the relative humidity was in no case a causative factor in the ocurrence of respiratory illness among school children. These observations on the hygiene of ventilation speak for themselves. Incidentally they give evidence of the value of one of the bureaus in the municipal health department organization of New York City which has recently become the target for something savoring of political interference.—*Journal A. M. A.*, August 3.

*Removal of Old Root Filling*

Generally in these cases there is only one cone in each canal, in which event acid will soon work its way around the cone, when it may be loosened with a suitable instrument and removed in one piece. However, occasionally it becomes necessary to remove a filling where the guttapercha has been packed firmly into the canal. Here the use of xylol or xylene will aid materially in disintegrating the guttapercha. While the agent does not dissolve the guttapercha in the sense that chloroform does, it readily attacks and disintegrates the material, making its removal comparatively easy after a few minutes.—J. P. Buckley, *Dental Summary*.

*Infallible Root Fillers* There are frequent suggestions of 100 per cent. successes following a rare combination of skill and method in treating and filling roots. If there is any human being for whom the divine attribute of infallibility can be justly claimed, I am quite sure in my own mind that he is not a member of the dental profession.—A. R. Starr, in *Journal N. D. A.*

*Interrupted General Anesthesia* At a recent meeting of the Société de chirurgie de Paris, Dr. Henri Chaput demonstrated a method of anesthesia consisting in the alternate administration and suppression of the anesthetic in the course of the operation. At the beginning of the procedure, the anesthetic (chloroform, ether, ethyl chlorid) is given in a quantity just sufficient to suppress sensibility and all reaction on the part of the patient. The anesthetic is then discontinued and the operation is proceeded with until the patient begins to react in such manner as to interfere with the progress of the work; then the anesthesia is resumed and continued until the patient is again in condition for the surgeon to proceed with the operation, and so on.

The advantages of this method are said to be: The corneal reflex is preserved; the face keeps the normal tint; the pupils are not modified to any extent; the pulse is strong and of good quality; the patient never vomits during the operation; cardiac or respiratory syncope never occur. As soon as the anesthesia is terminated, the patient regains consciousness almost immediately; the face is of good color and the features are calm. There is never any postoperative vomiting, sickness or shock; no icterus; and if it is a case of operation above the umbilicus the patient can get up the same day and go home. Chaput has used this method of anesthesia in more than one hundred cases without any untoward accident.—Paris letter to *Journal A. M. A.*, August 3, 1908.

*Oral Sepsis and Digestive Apparatus* Barker calls attention to the fact that serious arthritis, osteitis, osteomyelitis, periostitis, myositis, embolic pneumonia, pleuritis, nephritis, ureteritis, myocarditis, endocarditis, pericarditis, lymphadenitis, neuritis, neuralgia, iritis, anemia, septicemia and pyemia may, in certain cases, have their origin in oral sepsis and there is a growing tendency to look on the same etiologic factor as contributory, in many instances, to chronic degenerative diseases like arteriosclerosis and arterial hypertension. In the origin of disease in the digestive apparatus the forms of oral sepsis principally concerned consist of two groups: (1) alveolar infections secondary to diseases of the dental pulp; and (2) gingivitis, pyorrhea alveolaris, and infections about impacted teeth and unerupted teeth. The first group includes: (a) acute peridontitis (apical, interradial or lateral), in which the tooth becomes tender, especially at night, and feels longer and looser than it should, so that mastication becomes painful, often resulting in an acute alveolar abscess, in an alveolar parulis (or flat swelling under the periosteum), or in a chronic alveolar abscess with a sinus to the gum or face; and (b) proliferative periodontitis, that leads to granuloma (apical, interradial or lateral), usually starting as a result of the devitalization of a tooth and imperfect treatment of the root canal, and causing no symptoms referable to the tooth or gums recognizable by the patient.

The condition is easily recognizable in roentgenograms made on intro-oral dental films; without such roentgenograms it can scarcely be recognized with certainty. Barker states that any person who has devitalized teeth in his mouth does well to have them roentgenographed at intervals in order that a developing proliferative periodontitis shall not be overlooked. The second group of oral infections includes, as stated, gingivitis, pyorrhea alveolaris, and infections about impacted teeth and unerupted teeth. Much toxic matter may be directly absorbed from such infection areas. Often pus is swallowed over long periods (especially in pyorrhea alveolaris), or local extensions or metastatic infections may occur. Inspection of the mouth, palpation of the gums, and roentgenographic examinations make the diagnosis clear. The commonest varieties of disturbance of the digestive apparatus associated with the forms of oral sepsis mentioned above are: (1) gastritis and gastro-enteritis, (2) achylia gastrica, (3) pylorospasm, and (4) a toxic hepatopathy. It seems probable also that some cases of (5) gastric and duodenal ulcer are secondary to oral sepsis. Whether (6) appendicitis and (7) cholecystitis may occasionally, as some think, be due to metastatic infection from a primary oral focus, Barker says, is still in doubt.—*Journal A. M. A.*, August 17, page 602.

*To Refit Vulcanite Denture Without Vulcanizing*

Smear the denture with plaster, place in the mouth and when hard remove. Pour a model. Remove the denture and plaster from this cast. Having had the shot of a Parker shot-swager in boiling water or glycerine, place the denture upon the cast, and embed with the hot shot in the swager. Do not hammer, but place a heavy weight upon the piston. Remove when cold.—L. E. Custer, *Dental Summary.*

*Danger in Pressure Anesthesia*

Dr. Endleman, in the *Pacific Gazette,* warns against the use of pressure anesthesia of infected pulps because of the danger of forcing infected serum into the periapical tissues, with conseqent serious infection and fatal results.—Why could not this be avoided by sterilizing the cavity and bleeding the pulp freely before applying the pressure, and discontinuing the pressure immediately when the patient indicates that he feels no sensation?—Editor, *Register.*

